# Assessment of glucose levels in pregnant women with history of COVID-19 in a case-control study

**DOI:** 10.3389/fphys.2022.988361

**Published:** 2022-09-16

**Authors:** Cécile Monod, Grammata Kotzaeridi, Daniel Eppel, Tina Linder, Latife Bozkurt, Irene Hösli, Christian S. Göbl, Andrea Tura

**Affiliations:** ^1^ Department of Obstetrics and Gynaecology, Medical University of Vienna, Vienna, Austria; ^2^ Department of Obstetrics, University Hospital Basel, Basel, Switzerland; ^3^ Department of Internal Medicine III, Clinic Hietzing, Vienna Health Care Group, Vienna, Austria; ^4^ CNR Institute of Neuroscience, Padova, Italy

**Keywords:** COVID-19, dysglycemia, gestational diabetes mellitus, oral glucose tolerance test, pregnancy, SARS-CoV-2

## Abstract

Severe Acute Respiratory Syndrome CoronaVirus 2 (SARS-CoV-2) infection may negatively affect glucose metabolism. This study aims to assess glucose levels, prevalence of gestational diabetes mellitus (GDM) and perinatal outcome in women with history of COVID-19. To this purpose, a group of 65 patients with history of COVID-19 and 94 control patients were retrospectively recruited among pregnant women who attended the pregnancy outpatient department between 01/2020 and 02/2022. Glucose data from an oral glucose tolerance test (OGTT), GDM status and obstetric complications were assessed. We observed no differences in average (*p* = 0.37), fasting (*p* = 0.62) or post-load glucose concentrations (60 min: *p* = 0.19; 120 min: *p* = 0.95) during OGTT. A total of 15 (23.1%) women in the COVID-19 group and 18 (19.1%) women in the control group developed GDM (*p* = 0.55). Moreover, caesarean section rate, weight percentiles and pregnancy outcomes were comparable between the groups (*p* = 0.49). In conclusion, in this study we did not identify a possible impact of COVID-19 on glucose metabolism in pregnancy, especially with regard to glucose concentrations during the OGTT and prevalence of GDM.

## Introduction

In pregnancy, Severe Acute Respiratory CoronaVirus 2 (SARS-CoV-2) infection may be associated with risk of adverse outcomes (Di Mascio et al., 2020; Vouga et al., 2021). In that condition, maternal comorbidities, such as pre- and/or gestational diabetes, confer particularly high risk for adverse pregnancy outcomes, and for severe progression of the SARS-CoV-2 infection (Vouga et al., 2021). Indeed, diabetes has been clearly associated with COVID-19 severity and increased mortality, as shown by several studies and summarized by reviews and meta-analyses (Barron et al., 2020; Kumar et al., 2020). In addition, new-onset of diabetes has been described in the course of SARS-CoV-2 infection, this suggesting a bidirectional relationship between COVID-19 and diabetes (Apicella et al., 2020; Eberle et al., 2021). However, possible relationships between COVID-19 and gestational diabetes mellitus (GDM), or, more generally, dysglycemia during pregnancy, has been scarcely investigated.

The aim of this study was therefore to assess glucose levels in pregnant women with and without history of COVID-19, as derived by the diagnostic 75-g oral glucose tolerance test (OGTT) in mid-gestation. Prevalence of GDM and perinatal outcome in women with history of COVID-19 were also assessed. To our knowledge, no previous study reported OGTT glucose data in pregnant women with and without COVID-19 history.

## Materials and methods

### Patients, experiments, measurements

This study was designed as a retrospective, open, mono-center case-control study. All pregnant women attending the pregnancy outpatient department (Department of Obstetrics and Gynaecology, Medical University of Vienna) between 01/2020 and 02/2022 with history of COVID-19 were included. Patient’s data were assessed from the patient’s charts including glucose data from the OGTT, GDM status, mode of delivery, body mass index (BMI) before pregnancy, and neonatal outcome. Calculations of neonatal age and sex adjusted percentiles were performed by using international anthropometric standards (Villar et al., 2014). Pregnant women were included to serve as control subjects if there was no mention of a SARS-CoV-2 infection in the patient’s chart. A woman was selected for the control group if she gave birth immediately before or after a woman who served as a case. Women were not included in the control group if they overlapped with a case, had missing OGTT data or met other exclusion criteria. For both patients’ groups, exclusion criteria were preconceptional diabetes (type 1, type 2 or others), preconceptional endocrine disorder (Cushing’s syndrome, Addison’s disease), history of bariatric surgery or other malabsorption diseases (Crohn’s disease, ulcerative colitis, coeliac disease), diagnostic OGTT not performed in the recommended time range (24+0 to 27+6 pregnancy weeks) or, for the COVID-19 group, OGTT performed before SARS-CoV-2 infection. A total of 159 pregnant women met the inclusion criteria. Of them, 65 women had history of COVID-19 (cases), whereas 94 women had no history of COVID-19 (controls). More precisely, 38 women had the infection during pregnancy, and 27 before (ranging from 1 year up to 2 weeks before pregnancy).

In accordance with the IADPSG criteria, GDM diagnosis was made if glucose levels at the OGTT exceeded the following thresholds: 92 mg/dl at fasting, 180 mg/dl at 1 h, 153 mg/dl at 2 h (International Association of Diabetes and Pregnancy Study Groups Consensus Panel et al., 2010; World Health Organization Guideline, 2014). Fasting glucose exceeding 126 mg/dl or 2-hour glucose exceeding 200 mg/dl was considered as a pre-existent diabetes (International Association of Diabetes and Pregnancy Study Groups Consensus Panel et al., 2010; World Health Organization Guideline, 2014). The study was approved by the Ethics Committee of the Medical University of Vienna, Austria (study 2272/2021), and performed in accordance with the Declaration of Helsinki.

### Statistical analysis

Categorical variables were summarized by counts and percentages, continuous variables data by median and interquartile range. Statistical comparison of glucose levels between the two groups was performed by Student’s t-test or rank based inference in case of skewed distribution, whereby normal distribution was evaluated by a graphical test. The association between SARS-CoV-2 infection and GDM status was assessed by binary logistic regression. An adjustment for demographic and anthropometric variables (e.g., pregestational BMI) was performed by using generalized linear models. Differences in more than two groups were assessed by analysis of variance and Fisher protected least significant difference test. *p*-values ≤ 0.05 (two-sided) were considered statistically significant. With a sample size of 65 patients and 94 control subjects we achieved a power of 90% to exclude a mean difference of 10 mg/dl in mean glucose during the OGTT, providing a standard deviation of 19 mg/dl for Student’s t-test. Analyses were performed in R (V4.0.2) and contributing packages (R Project for Statistical Computing, 2022).

## Results

**FIGURE 1:**
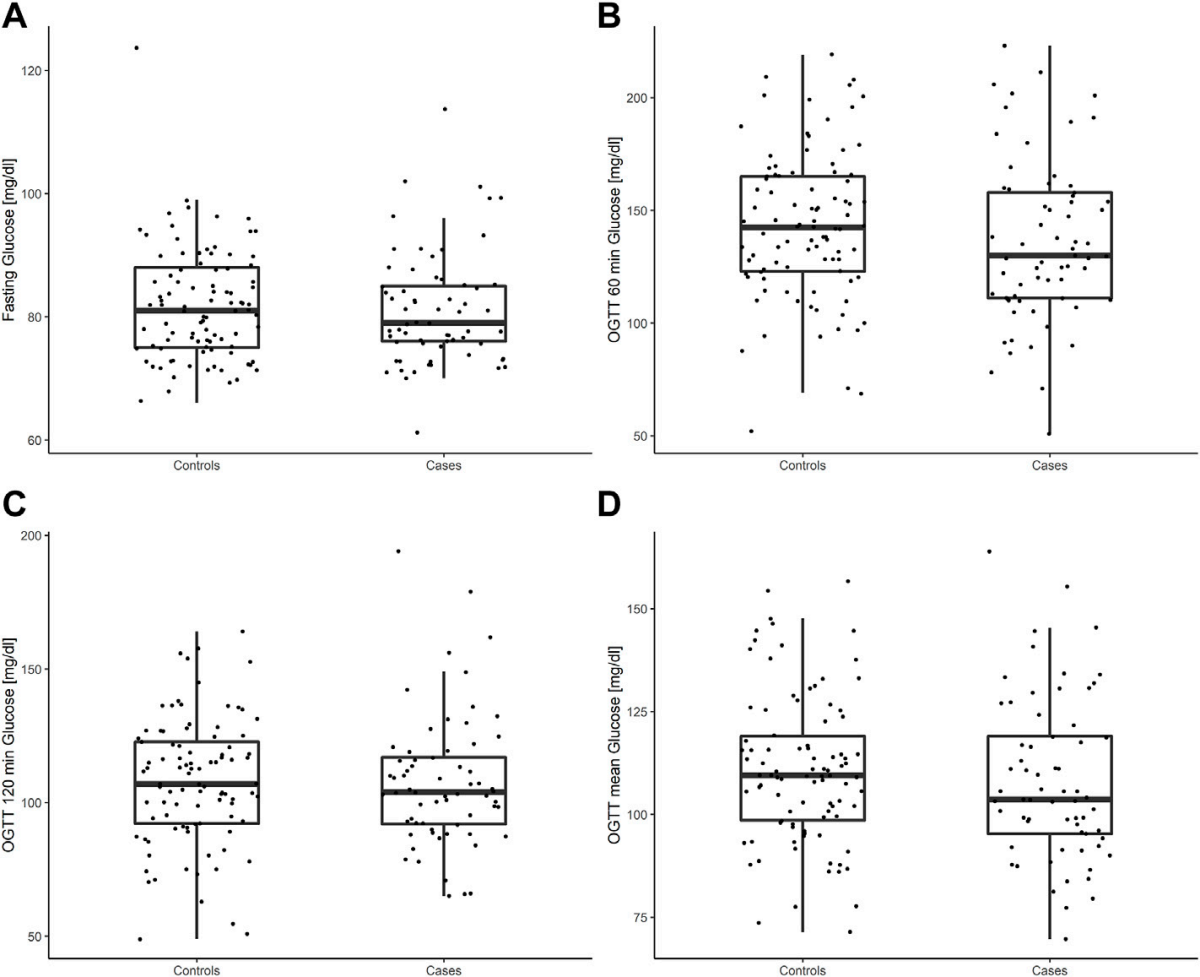
Glucose concentrations during the diagnostic 75-g OGTT at 24–28 weeks of gestation in women with history of SARS-CoV-2 infection (cases) and the control group without infection (controls): fasting glucose **(A)**, 60 min post load glucose **(B)**, 120 min post load glucose **(C)**, mean glucose **(D)**.

The characteristics of the study population, including OGTT glucose levels, are presented in Table 1. There were no differences between cases and controls in mean glucose concentrations (mean difference: -2.7, 95% CI -8.7 to 3.2 mg/dl, *p* = 0.37). Comparable results were also observed for fasting glucose (mean difference: -0.72, 95% CI -3.6 to 2.2 mg/dl, *p* = 0.62), glucose at 60 min (mean difference: -7.2, 95% CI -18.2 to 3.6 mg/dl, *p* = 0.19) and at 120 min (mean difference: -0.2, 95% CI -7.9 to 7.4 mg/dl, *p* = 0.95; see also Figure 1). A total of 15 (23.1%) women in the COVID-19 group and 18 (19.1%) women in the control group developed GDM (*p* = 0.55). There were no differences in the proportions of GDM women with need of glucose lowering medications between groups.

**TABLE 1:** Maternal characteristics, OGTT data, obstetrical and neonatal outcomes for the group of women with history of SARS-CoV-2 infection (cases) and the group without infection (controls).

	Cases	Controls	*p*-value
	(*n* = 65)	(*n* = 94)	
Age (years)	31.7 ± 5.8	31.6 ± 6.0	0.94
Parity	1 (0–2)	0 (0–2)	0.22
BMI, before pregnancy (kg/m^2^)	25.2 ± 4.9	24.2 ± 5.3	0.24
Multiple pregnancy (n,%)	4 (6.2)	14 (14.9)	0.09
Fasting glucose (mg/dl)	81.1 ± 9.2	81.8 ± 9.1	0.62
OGTT glucose, 60 min (mg/dl)	135.9 ± 36.2	143.1 ± 32.8	0.19
OGTT glucose, 120 min (mg/dl)	107.6 ± 24.7	107.8 ± 23.6	0.95
OGTT glucose mean (mg/dl)	108.2 ± 19.6	110.9 ± 18.2	0.37
GDM (n, %)	15 (23.1)	18 (19.1)	0.55
Pharmacotherapy in GDM (n, %)	6 (40.0)	11 (57.9)	0.30
GA delivery (weeks)^*^	39.6 (38.7–40.3)	39.1 (38.4–40.1)	0.19
APGAR 1 min^*^	9 (9–9)	9 (9–9)	0.79
APGAR 5 min^*^	10 (10–10)	10 (10–10)	0.64
APGAR 10 min^*^	10 (10–10)	10 (10–10)	0.08
Caesarean section (n, %)^*^	24 (40.7)	28 (35.0)	0.49
Birth weight (kg)^*^	3.34 ± 0.58	3.34 ± 0.58	0.99
Birth weight (pct)^*^	59.8 ± 29.1	59.8 ± 31.0	0.99

Data are mean ± SD or median (IQR) and count (%).

* Twin pregnancies were excluded from this analysis.

BMI, body mass index; OGTT, oral glucose tolerance test; GDM, gestational diabetes mellitus; GA, gestational age; APGAR, Appearance, Pulse, Grimace, Activity, Respiration.

Furthermore, we identified 27 (41.5%) with infection before pregnancy, 25 (38.5%) with infection in first (<14+0 weeks) and 13 (20%) with infection in second and early third trimester (14+0–27+6 weeks). However, there were no differences in the incidence of GDM (*p* = 0.524) or glucose values during the OGTT observed between these groups (fasting: *p* = 0.702; 60 min: *p* = 0.589; 120 min: *p* = 0.253).

After excluding 18 patients with twin pregnancies, the rate of caesarean section and the weight percentiles were comparable between the groups (35 vs. 41%, *p* = 0.49, 60 ± 31 vs. 60 ± 29, *p* = 0.99, respectively). Details are provided in Table 1. Further neonatal outcomes are available in the supplementary material (Supplementary Table S1).

## Discussion

Several previous studies addressed the relationship between SARS-CoV-2 infection and type 2 diabetes (Barron et al., 2020; Kumar et al., 2020). In contrast, possible relationships between SARS-CoV-2 infection and gestational diabetes, or dysglycemia during pregnancy, are scarce. Especially, to our knowledge, no previous study presented OGTT glucose data in pregnant women with history of COVID-19 as compared to women without COVID-19 history, and this is a merit of our study. Our analysis showed comparable glucose levels during the diagnostic OGTT at mid-gestation, as well as similar prevalence of GDM, in pregnant women with and without COVID-19 history.

In pregnant women with gestational or pre-existing diabetes, the risk for a severe course of SARS-CoV-2 infection is increased compared to pregnant women without diabetes (Vouga et al., 2021). Furthermore some authors described an increased incidence of new-onset diabetes during the course of SARS-CoV-2 infection, though the exact pathophysiological bidirectional relationship between SARS-CoV-2 infection and diabetes is not yet fully understood (Apicella et al., 2020; Eberle et al., 2021). It is hypothesized that a surface protein of SARS-CoV-2 virus binds to the angiotensin converting enzyme 2 (ACE2) receptor and allows the virus to enter cells. The ACE2 receptor is expressed on the surface of several essential metabolic organs, and in particular on pancreatic beta cells. This may lead to beta-cell dysfunction and explains the worsening of metabolic state observed in SARS-CoV-2 infected patients with overt diabetes (Yang et al., 2010). This mechanism may also explain the higher rate of new onset diabetes during the course of COVID-19. Likewise, different viral infections (such as hepatitis C) can lead to type 2 diabetes by disturbing beta-cell function, or can even trigger type 1 diabetes onset (Apicella et al., 2020; Eberle et al., 2021). These aspects may be of particular importance for pregnant women, whose insulin sensitivity is already physiologically decreased (Göbl et al., 2015).

Due to these pathophysiological mechanisms and observations, we were expecting higher glucose levels during the OGTT, as well as increased prevalence of GDM in pregnant women with history of SARS-CoV-2 infection than in those without. However, this was not observed in our study. Possible explanations are the limited number of pregnant women, though on the other hand, it is worth noting that we had a high power (90%) to exclude a clinically relevant difference in OGTT mean glucose concentrations equal or higher than 10 mg/dl. Furthermore, it is also worth noting that another study showed no association of GDM and COVID-19 diagnosis, at least in those women who were at normal weight and did not use insulin (Eskenazi et al., 2021), and this appears substantially consistent with our results.

On the other hand, a prospective case-control study by Radan et al. found a higher GDM incidence in women with SARS-CoV-2 infection as compared to a historical control-group (Radan et al., 2022). However, this study has some substantial methodological differences compared with ours. Especially, women who gave birth before the beginning of the pandemic were included in the control-group, and it is known that a global increased incidence of GDM has been observed during the pandemic, possibly due to lifestyle changes (Cauldwell et al., 2021; Sun et al., 2021; Kleinwechter et al., 2022; Zanardo et al., 2022). This may explain the diverging findings. Of note, in the study by Radan et al. (2022), SARS-CoV-2 infection was considered both before and after GDM diagnosis, and the subgroup analysis showed comparable GDM incidences regardless of the timepoint of infection, supporting the hypothesis of a bidirectional relationship between GDM and COVID-19 (Radan et al., 2022). Nonetheless, we believe that it remains not fully elucidated whether the timing of the infection in the course of pregnancy may play a role in the development of impaired glucose regulation. In addition, it is currently not known to which extent disturbances in glucose metabolism are reversible over time. It should also be mentioned that worse maternal outcomes for SARS-CoV-2 infection (at the same time or shortly after GDM diagnosis) were reported in one of the previous studies (Kleinwechter et al., 2022).

While we could not identify significant differences in cases with SARS-CoV-2 infection during versus before pregnancy, this aspect needs to be addressed by larger registry studies. In fact, our investigation should be deemed as a pilot study, likely able to trigger further studies in the field. Nonetheless, on the base of the current evidence as provided by our analysis, from a clinical point of view the key message of the study appears being that SARS-CoV-2 infection does not cause specific concerns related to glucose tolerance in pregnancy. Indeed, even in women with overt GDM, SARS-CoV-2 infection was not associated to further deterioration of the glucose tolerance, as the proportion of women with need of glucose lowering medication was comparable. Thus, SARS-CoV-2 infection in pregnancy, even when complicated by GDM, may not call for reinforced care in terms of dysglycemia treatment. Future longitudinal studies will have to address these aspects as well.

We summarize that we did not identify a significant impact of SARS-CoV-2 infection on glucose levels in pregnancy. Especially, we found no differences in OGTT glucose concentrations and GDM prevalence or pregnancy outcomes between pregnant women with and without history of COVID-19. Possible long-term consequences in metabolic health for both mothers and offspring need to be addressed in future studies.

## Data Availability

The raw data supporting the conclusions of this article will be made available by the authors, without undue reservation.
