# Supplementary material for: Assessment of glucose levels in pregnant women with history of COVID-19 in a case-control study
Source: Front Physiol. 2022 Sep 16;13:988361. doi: 10.3389/fphys.2022.988361 (PMC9522974; doi:10.3389/fphys.2022.988361)
Supplement: Supplementary file 1 [file Table1.docx]

**Table S1 Additional Neonatal outcomes**

|  | Cases | Controls | p-value |
| --- | --- | --- | --- |
| NICU transfer (%) | 9 (13.8) | 8 (8.5) | 0.285 |
| Prematurity (<37 weeks) (%) | 10 (15.4) | 15 (16.0) | 0.922 |
| Indication for NICU   - Hypoglycaemia - Prematurity singleton - Prematurity twin - RDS - Cardiac malformations - Unknown | 0  2  2  3  2  0 | 0  1  4  0  2  1 |  |

Twin pregnancies are included. NICU: neonatal intensive care unit, RDS: respiratory distress syndrome
